# An Integrated Strategy for Rapid Discovery and Identification of Quality Markers in Gardenia Fructus Using an Omics Discrimination-Grey Correlation-Biological Verification Method

**DOI:** 10.3389/fphar.2021.705498

**Published:** 2021-06-24

**Authors:** Rong Dong, Qingping Tian, Yongping Shi, Shanjun Chen, Yougang Zhang, Zhipeng Deng, Xiaojing Wang, Qingqiang Yao, Liwen Han

**Affiliations:** ^1^ School of Pharmacy and Pharmaceutical Science, Shandong First Medical University and Shandong Academy of Medical Sciences, Jinan, China; ^2^ School of Pharmaceutical Science, Shanxi Medical University, Taiyuan, China; ^3^ Taiyuan Maternity and Child Health Care Hospital, Taiyuan, China; ^4^ College of Pharmacy, Shandong University of Traditional Chinese Medicine, Jinan, China

**Keywords:** gardenia fructus, zebrafish, quality markers, herbal metabolomics, gray correlation analysis, anti-inflammatory, liver protection

## Abstract

**Background:** Gardenia Fructus (GF), a traditional Chinese medicine of *Gardenia Ellis* in Rubiaceae family, has the potential to clear heat and purge fire and has been widely used to treat multiple infection-related diseases. However, the quality markers (Q-Markers) of GF have not been revealed comprehensively.

**Methods:** In this experiment, the transgenic zebrafish lines, *Tg (l-fabp:EGFP)* and *Tg (lyz:EGFP),* were used to evaluate two main kinds of traditional efficacies of GF, hepatoprotective and anti-inflammatory effects. All the GF samples from different production areas were tested their anti-liver injury and anti-inflammantory activities. High-performance liquid chromatography-quadrupole time-of-flight mass spectrometry method (HPLC-Q-TOF/MS) was employed for herbal metabonomic analysis of GF samples. Gray correlation analysis (GCA) was utilized to screen out the components closely associated with the activities. Finally, the zebrafish model was applied to verify the bioactivity of the crucial components to determine the Q-Markers of GF.

**Results:** The zebrafish models were established by inducing with hydrogen peroxide or copper sulfate and applied to quickly evaluate the hepatoprotective effect and inflammation of GF samples. 27 potentially active components for liver protection and 21 potentially active components with anti-inflammatory properties were identified by herbal metabolomic analysis based on HPLC-Q-TOF/MS. The GCA result showed that five of the 27 components were highly correlated with liver protection, 15 of the 21 components were highly correlated with anti-inflammatory activity. Among them, geniposide and crocin-1 were confirmed their bioactivities on zebrafish experiment to be responsible for the protective effects of GF against liver injury, and genipin-1-β-D-gentiobioside, quinic acid, gardenoside, d-glucuronic acid, l-malic acid, mannitol, rutin, and chlorogenic acid were confirmed to be responsible for the anti-inflammatory effects. Finally, according to the screening principles of Q-Markers, genipin-1-β-D-gentiobioside, geniposide, and gardenoside were preliminarily identified to be the Q-Markers of GF.

**Conclusion:** This study established an effective research strategy of “Omics Discrimination-Grey Correlation-Biological Verification,” which enabled the rapid identification of key pharmacological components of GF. These markers have provided a scientific basis for constructing a modern quality evaluation system for GF.

## Introduction

Gardenia Fructus, the dried fruit of *Gardenia jasminoides* Ellis in Rubiaceae family, has been recorded in the *Shennong Bencaojing treatize* of the Han Dynasty (BC 202–AD 220) in China. GF has the potential to clear *Heat* and purge *Fire* (Chen et al., 2020). Furthermore, GF plays a significant clinical role in the prevention and treatment of chronic diseases such as liver and gallbladder diseases (Fan et al., 2020) and metabolic diseases (L. Wang et al., 2020). Besides, GF has been listed in medicine food homology in China, and is also a common traditional medicinal food for the health industry. However, the existing quality standard system of traditional Chinese medicine is far from the requirements of the actual market circulation and authentic variety evaluation (X.Wu et al., 2018; C. Liu, Guo. DA and L. Liu et al., 2018). Hence, studying the quality evaluation chemical makers is crucial for ascertaining the intrinsic quality of GF.

Quality markers (Q-Markers) is a new concept of scientific quality evaluation of TCM proposed by the Chinese academician Changxiao Liu in 2016. Q-markers is a chemical substance inherent or formed during processing and preparation of Chinese medicine materials and TCM products (such as Chinese herbal pieces, Chinese herbs decoction, extraction of TCM, Chinese patent medicine preparation). It is closely related to the functional properties of TCM, and is used as a marker substance reflecting the safety and effectiveness of TCM for quality control (C. X. Liu et al., 2016), and also forms the basis of quality evaluation (Ren et al., 2020). In this research, we hope to explore the Q-Markers of GF based on this theory, which can lay the foundation for the establishment of scientific quality control methods for GF.

For the quality evaluation of TCM, the use of Q-Markers is an internationally accepted method to reflect the efficacy and quality by assessing a limited number of chemical components. However, it is not easy to confirm these key chemical components (L. Xu et al., 2018). The routine research methods are usually hard to track the unknown active chemical components, or vast samples produced from separation and separation process (X. Liu et al., 2021). Therefore, more and more researchers are attempting to explore and develop efficient identification methods for key medicinal components. Metabolomics is an important branch of systematic biology, which has high resolution and extremely low detection limit. This approach can simultaneously detect integral components in TCM (Jiang et al., 2016). However, due to the large number of chromatographic peaks and small sample size, it is not sufficient to perform most of the general pharmacological tests. Therefore, we endeavored to propose a more effective method to improve the accuracy of chemical components extracted from the complex chemical system of TCM.

At present, models of liver protection and inflammation based on rodents (rats and mice) are commonly used for evaluating the efficacy of TCM (Chiang et al., 2020; C. Y.; Li et al., 2016; W. K.; Li et al., 2019). However, a large amount of experimental samples volume is required for *in vivo* models, which is not easy to be achieved for sample extracts of TCM. Moreover, it is difficult to realize the evaluation of the monomer components in the later stage. Zebrafish (*Danio rerio*), a tropical freshwater fish of the Cyprinidae family, is a vertebrate model and is often used to study human diseases (Lieschke and Currie, 2007). When compared with mice and other commonly used *in vivo* animal models, zebrafish has the advantages of small size, large number of offspring, high transparency, easy breeding, low feeding cost, and the ease of carrying out large-scale research (Lieschke and Currie, 2007). The function of liver cells and histopathological changes in zebrafish are very similar to humans. The molecular mechanism of the development is also consistent with that of mammals (Goessling and Sadler, 2015). Zebrafish is also widely used to study the immune response to the acute phase of infection, the interaction between pathogens and the host, and the real-time migration of injured immune cells (Torraca and Mostowy, 2018). Therefore, zebrafish was chosen as an experimental model in this study. Although the limited activities could hardly represent the efficacy of GF, we believe that these two activities reflect main parts of the efficacy of GF. What’s more, the zebrafish experiment supplied considerable convenience to verify the activities of the selected monomer components *in vivo* and validate the screening results (Y. P. Shi et al., 2020).

Finally, we proposed a new integrated research strategy, namely the “Omics Discrimination-Grey Correlation-Biological Verification” by using herbal metabolomics, zebrafish model, and other technologies. Exploration of the pharmacodynamic markers closely related to the efficacy of GF provides an important basis for constructing a quality evaluation system with Q-Markers as the core.

## Materials and Methods

### Chemicals, Drugs, and Reagents

Hydrogen peroxide (30%, CAS:7722-84-1, Lot#20160908), ethanol (analytical grade 64-17-5), and formic acid (analytical grade, CAS:64-18-6) were acquired from Sinopharm Chemical Reagent Co., Ltd. (Shanghai, China); Acetonitrile (HPLC grade, CAS:75-05-8) was purchased from Oceanpak (Goteborg, Sweden). Tricaine (CAS:886-86-2) were purchased from Sigma-Aldrich Co.; dimethyl sulfoxide (DMSO, CAS:67-68-5, Lot#39075) was purchased from Sangon Biotech Co.; Ibuprofen (CAS:15687-27-1, Lot#VM9V-S7BU); cupric sulfate (CAS:7758-99-8); Ultrapure water was supplied by Watsons Ltd. (Hong Kong, China); Genipin-1-β-D-gentiobioside (CAS:29307-60-6, Lot#P20N8F48431), geniposide (CAS:24512-63-8, Lot#C1722062), crocin-1 (CAS:42553-65-1, Lot# P01N8F45675), quinic acid (CAS:77-95-2, Lot#K29D9B77039), gardenoside (CAS:54835-76-6, Lot#P160A10F95425), d-glucuronic acid (CAS:556-12-3, Lot# K14M10S82777), l-Malic acid (CAS:97-67-6, Lot#S24O10I101046), mannitol (CAS:87-78-5, Lot#SM0513GB13), rutin (CAS:153-18-4, Lot#T10J10Z90356), chlorogenic acid (CAS:327-97-9, Lot#Y22M8K36544), Shanzhiside (CAS:29836-27-9, Lot#P30J9F64637), d-glucaric acid (CAS:87-73-0, Lot#P23B15G75287) were provided by Shanghai Yuanye Bio-Technology Co., Ltd. The purity of all the chemicals were greater than 98%.

The sources and the time of collection of dried ripe fruits of GF were indicated (Table 1). All GF samples were identified as the dried fruits of *Gardenia jasminoides* Ellis belonging to the Rubiaceae family by Dr Liwen Han, Associate Professor, Shandong First Medical University.

**TABLE 1:** Information related to GF samples.

Sample no	Producing area	Collected time
S1	Huoshan, Anhui	September 2018
S2	Kunming, Yunnan	September 2018
S3	Jian, Jiangxi	September 2018
S4	Panan, Zhejiang	September 2018
S5	Deqing, Zhejiang	September 2018
S6	Jichun, Hubei	September 2018
S7	Jiujiang, Jiangxi	September 2018
S8	Fuding, Fujian	September 2018
S9	Fushun, Jiangxi	September 2018
S10	Jiujiang, Jiangxi	November 2018
S11	Jiujiang, Jiangxi	December 2018
S12	Jiujiang, Jiangxi	October 2019
S13	Fuding, Fujian	October 2018
S14	Fuding, Fujian	November 2018
S15	Fuding, Fujian	October 2019
S16	Fuding, Fujian	November 2019
S17	Fuding, Fujian	September 2019
S18	Panan, Zhejiang	October 2018
S19	Panan, Zhejiang	November 2018
S20	Panan, Zhejiang	October 2019
S21	Panan, Zhejiang	November 2019
S22	Jichun, Hubei	October 2018
S23	Jichun, Hubei	November 2018
S24	Jichun, Hubei	October 2019
S25	Jichun, Hubei	November 2019
S26	Jichun, Hubei	September 2019

### Zebrafish Maintenance and Embryo Collection

The transgenic line zebrafish, *Tg (l-fabp:EGFP)* and *Tg (lyz:EGFP)*, were provided by Shandong Province Engineering Research Center of Zebrafish Models for Human Diseases and Drug Screening (Biology Institute of Shandong Academy of Sciences). The animals were cultivated in a professional breeding system (Shanghai Haisheng Biotech Co., Ltd.) and maintained under a 14 h/10 h light/dark cycle at a constant temperature of 28 ± 0.5°C. The zebrafish were fed with fairy shrimp twice a day at 9:00 and 16:00 h. Adult male and female zebrafish were placed in a ratio of 2:2 on both sides of a partition board in a mating tank at night; the divider was removed at 8:00 h the next day. The zebrafish was stimulated by light to mate and lay eggs naturally. The embryos were collected within 1 h of spawning and rinsed thrice with fresh water. The clean embryos were transferred to Petri dishes containing fish water (5 mM NaCl, 0.17 mM KCl, 0.4 mM CaCl_2_, and 0.16 mM MgSO_4_) and incubated at 28.5°C for subsequent experiments (Avdesh. A et al., 2012). The animal study was reviewed and approved by Ethics Committee of Shandong First Medical University, Shandong First Medical University & Shandong Academy of Medical Sciences*.*

### Preparation of Sample Solutions

All GF samples were ground to a powder form and sifted through a 60-mesh screen. Then, the powder was extracted twice using 70% ethanol with a material-to-liquid ratio of 1:8 for 1 h. The supernatant of each sample was collected after centrifugation at 5,000 r/min for 10 min. The extracted GF samples were obtained by rotary evaporation and drying at 60°C. Anesthetic (tricaine) solution of 1 g L^−1^ concentration was prepared with distilled water; 30% hydrogen peroxide was diluted 60 times with ultrapure water to make a solution of 5 ml L^−1^ concentration; CuSO_4_.5H_2_O was made into a mother liquor of 5 g L^−1^ concentration with distilled water; Ibuprofen was dissolved in dimethyl sulfoxide (DMSO) to prepare a solution of 4 g L^−1^ concentration; gardenoside, genipin-1-β-D-gentiobioside, crocin-1, quinic acid, d-glucuronic acid, l-malic acid, mannitol, rutin, chlorogenic acid, shanzhiside, and d-glucaric acid were each prepared into stock solutions of 100 g L^−1^ concentration with DMSO. Each GF sample (for HPLC-Q-TOF/MS analysis) was made a solution of 1 g L^−1^ concentration with acetonitrile water (5:95, v/v). Gardenoside, genipin-1-β-D-gentiobioside, crocin-1, quinic acid, d-glucuronic acid, l-malic acid, mannitol, rutin, and chlorogenic acid were accurately weighted and dissolved in 25 ml of acetonitrile water (5:95, v/v) through a microporous filter membrane to prepare sample solutions of the required concentration for the experiments one by one.

### Liver Protection and Anti-Inflammatory Experiments in Zebrafish

#### Experimental Design

Zebrafish larvae that were 3 days post-fertilization (dpf) were taken in a Petri dish. Larvae of normal and consistent development were selected and placed in a 6-well microplate, with 20 larvae per well. The animals was divided into liver protection group and anti-inflammatory group. All groups were pre-protected for 6 h. The control group (Con) was given only 0.5% DMSO. The indicated concentration gradient of the drug group was set as shown in the results section. In the liver protection experiment, all groups (excluding the control group) were treated with H_2_O_2_ at a concentration of 25 μL L^−1^ for 2 days to induce liver injury (Ma. J et al., 2020). The inflammation model of zebrafish was established by using CuSO_4_ at a concentration of 4 mg L^−1^ for 1 h (Gui et al., 2020). The positive group was treated with ibuprofen at a concentration of 4 mg L^−1^.

#### Image Capture and Statistical Analysis

Ten zebrafish larvae were randomly selected from each group for photographic observation. In the liver protection experiment, the fluorescence of liver was observed using a fluorescence microscopy. The images were collected and recorded for each zebrafish larva. Fluorescence intensity of the liver (% of control) was measured using Image Pro Plus 5.1 (Media Cybernetios Inc., Rockville, MD, United States). In the anti-inflammatory experiment, abnormalities and the distribution of the neutrophils were observed using a fluorescence microscope. The neutrophils migrating to the lateral neuromast and above were statistically analyzed by GraphPad Prism 6.01 (Graphpad Software Inc., La Jolla, CA, United States). One-way analysis of variance way was used to determine the statistical significance of the differences among the groups, and the experimental data were expressed as mean ± standard error (SE). The difference was considered to be significant when *p* was <0.05, and the difference was considered to be extremely significant when *p* was <0.01 (^##^ *p < 0.01* is vs. the control group; *p < 0.05 and **p < 0.01 are vs. model group).

### Herbal Metabolomic Analysis

#### HPLC-Q-TOF/MS Analysis of GF

HPLC-Q-TOF/MS consists of a liquid chromatography system (Agilent 1,200 Infinity Series) with a binary pump, degassing device, automatic sampler, and ultraviolet detector as well as a Q-TOF mass spectrometer (Agilent 6,520 series) to examine the GF samples. An Eclipse XDB-C18 column (4.6 × 250 mm; 5 μm; Agilent) was used as the chromatographic column. The chromatographic conditions were as follows: the mobile phase was composed of formic acid–water (0.1:100, v/v) (A) and acetonitrile (B). The gradient elution program was 7–20% B for 0–10 min, 20–30% B for 10–13 min, 30% B for 13–17 min, 30%–7% B for 17–18 min, and 7% B for 18–25 min at a flow rate of 1 ml/min. The sample injection volume was 20 μL. The temperature of the column was set at 25°C. The detection wave was set at 238 nm. The MS conditions were as follows: Electrospray ionization was in the negative ion mode, split ratio was 2:1, scan range m/z was 100–1,100 Da, nebulizer pressure was two psig, gas temperature was 350°C, drying gas flow rate was 12 L/min, and capillary voltage was 3500 V. The raw data of all GF samples were analyzed with Agilent MassHunter Workstation and R software (R Development Core Team, 2011).

#### Data Analysis

Principal component analysis (PCA) was used to describe the natural distribution and aggregation states of the samples (Nie et al., 2020). Orthogonal partial least squares-discriminant analysis (OPLS-DA) was employed to distinguish the different kinds of compounds from each other (Chung et al., 2019; B. M.; Huang et al., 2018). S-plots provided visible OPLS-DA for model explanation (Feng et al., 2018); A permutations test (PT) evaluated the credibility of the OPLS-DA model (Bocca et al., 2021) using the software SIMCA-P14.1.

### Gray Correlation Analysis

Gray correlation analysis (GCA) was used to examine the correlation between two groups of variations (P. Li et al., 2020). In this study, the signal intensities of the different components present in the 9 GF samples (S1-S9) obtained from different producing areas were measured. GCA was applied to investigate the spectral-effect relationships between signal intensity and biological activities related to liver injury and inflammation in the zebrafish.

Specifically, ${\text{x}}_{0}=\left\{{\text{x}}_{\text{0}}\left(\text{k}\right)|\text{k}=1,2,\ldots ,\text{n}\right\}=\left({\text{x}}_{\text{0}}\left(1\right),{\text{x}}_{\text{0}}\left(2\right),\ldots ,{\text{x}}_{\text{0}}\left(\text{n}\right)\right)$ was set as the sequence of system behavior characteristics. Furthermore, ${\text{x}}_{\text{i}}=\left\{{\text{x}}_{\text{i}}\left(\text{k}\right)|\text{k}=1,2,\ldots ,\text{n}\right\}=\left({\text{x}}_{\text{i}}\left(1\right),{\text{x}}_{\text{i}}\left(2\right),\ldots ,{\text{x}}_{\text{i}}\left(\text{n}\right)\right)$ was set as the sequence of system associated factors, where k refers to the samples collected from different origins, and i refers to the compounds. Subsequently, the correlation coefficient (k)) and the degree $\left({\text{r}}_{0\text{i}}\right)$ of GCA were formulated as follows:

$$\text{ε}\left(\text{k}\right)=\frac{\underset{\text{i}}{\min\;}\underset{\text{k}}{\min}\;\left|{\text{x}}_{0}\left(\text{k}\right)-{\text{x}}_{\text{i}}\left(\text{k}\right)\right|+\text{ ρ}.\underset{\text{i}}{\max\;}\underset{\text{k}}{\max}{\text{ |x}}_{0}\left(\text{k}\right)-{\text{x}}_{\text{i}}\left(\text{k}\right)}{\left|{\text{x}}_{0}\left(\text{k}\right)-{\text{x}}_{\text{i}}\left(\text{k}\right)\right|+\text{ ρ}.\underset{\text{i}}{\max\;}\underset{\text{k}}{\max}{\text{|x}}_{0}\left(\text{k}\right)-{\text{x}}_{\text{i}}\left(\text{k}\right)},$$

$${\text{r}}_{0\text{i}}=\frac{1}{\text{m}}\sum_{\text{k}=1}^{\text{m}}\text{ε}\left(\text{k}\right),$$

ρ is the resolution coefficient ranging from 0 to 1, usually 0.5.

The fluorescence intensity of the liver and the number of neutrophils spreading to the lateral neuromast of the zebrafish in the 9 GF samples were set as the sequence of system behavior characteristics. The signal intensities of different component variations of the 9 GF samples were set as the sequence of system associated factors. Owing to the different dimensions of the two datasets, dimensionless processing was performed on the original data to eliminate the adverse effects of unit inconsistencies. Especially, for dimensionless processing, each data point was divided by the average of the corresponding sequence to calibrate and calculate the correlation coefficients (M. Liu et al., 2020).

## Result

### Biological Activities of the GF Samples on the Zebrafish

**FIGURE 1:**
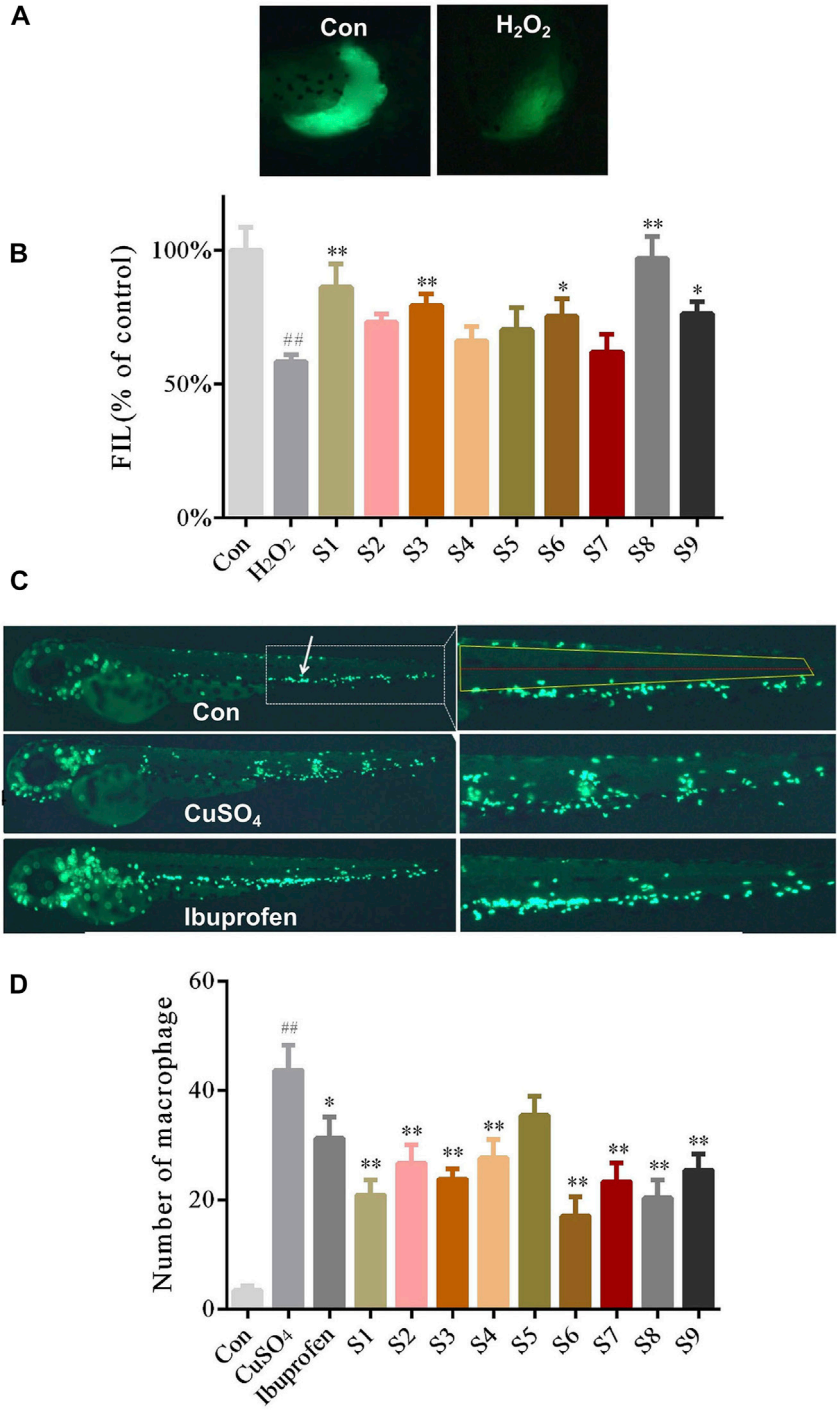
The protective effect of nine samples of GF (S1-S9) collected from different areas on liver injury and inflammation **(A)** Phenotypic micrograph of the transgenic zebrafish with fluorescence label in the liver **(B)** Statistical results of the fluorescence intensity of the liver in zebrafish until five dpf in all groups. *n* = 10 **(C)** Distribution phenotypic micrograph of neutrophils (white arrows) in zebrafish and enlarged drawing of neutrophils spreading to the lateral neuromast of the tail **(D)** Statistical results of the number of neutrophils diffusion in zebrafish in all groups, *n* = 10.

The zebrafish model of liver injury was produced using H_2_O_2_ at a concentration of 25 mgL^−1^ for 2 days. As shown in Figure 1A, the fluorescence intensity decreased in the liver of zebrafish in the H_2_O_2_ model group when compared with those in the control group. However, the fluorescence intensity in the zebrafish liver was significantly improved in the GF groups of S1, S3, S6, S8, and S9 (the dose received by each group was 500 mg L^−1^) compared to the H_2_O_2_ model group (Figure 1B). The results signify that the liver injury was relieved in the above GF groups. However, the 4 GF groups of S2, S4, S5, and S7 did not differ from the model group in a statistically significant manner. The two groups with the largest difference were S7 (Jiujiang, Jiangxi) and S8 (Fuding, Fujian), with liver fluorescence intensities of 62.0 and 97.0%, respectively.

Zebrafish models of inflammation were established with CuSO_4_ at a concentration of 4 mgL^−1^ for 1 h. As shown in Figure 1C, the right panel shows the magnification of the white-dashed box, whereas the yellow box represents the neutrophils diffusion region and the red line represents the lateral neuromast, at 32× magnification. the fluorescent spots near the lateral neuromast of the zebrafish tail were higher in the CuSO_4_ model group when compared with the control group. The number of fluorescent spots near the lateral neuromast of the zebrafish tail were significantly lowered after treatment with ibuprofen. The above results suggest that the inflammatory model of the zebrafish can be used to study the anti-inflammatory efficacy of GF. Neutrophils spreading close to the lateral neuromast of the zebrafish tail were remarkably inhibited in the GF groups of S1,S2, S3, S4,S6, S7, S8, and S9 (the dose received by each group was 500 mg L^−1^) when compared with the CuSO_4_ model group (Figure 1D). The average number of fluorescent spots near the lateral neuromast in the zebrafish tail in the S4 (Panan, Zhejiang) and S6 (Jichun, Hubei) groups were 27 and 17, respectively, and they exhibited the most significant statistical difference among all the GF treatment groups.

### Comparison of Activity in GF Samples Collected From Specific Locations

**FIGURE 2:**
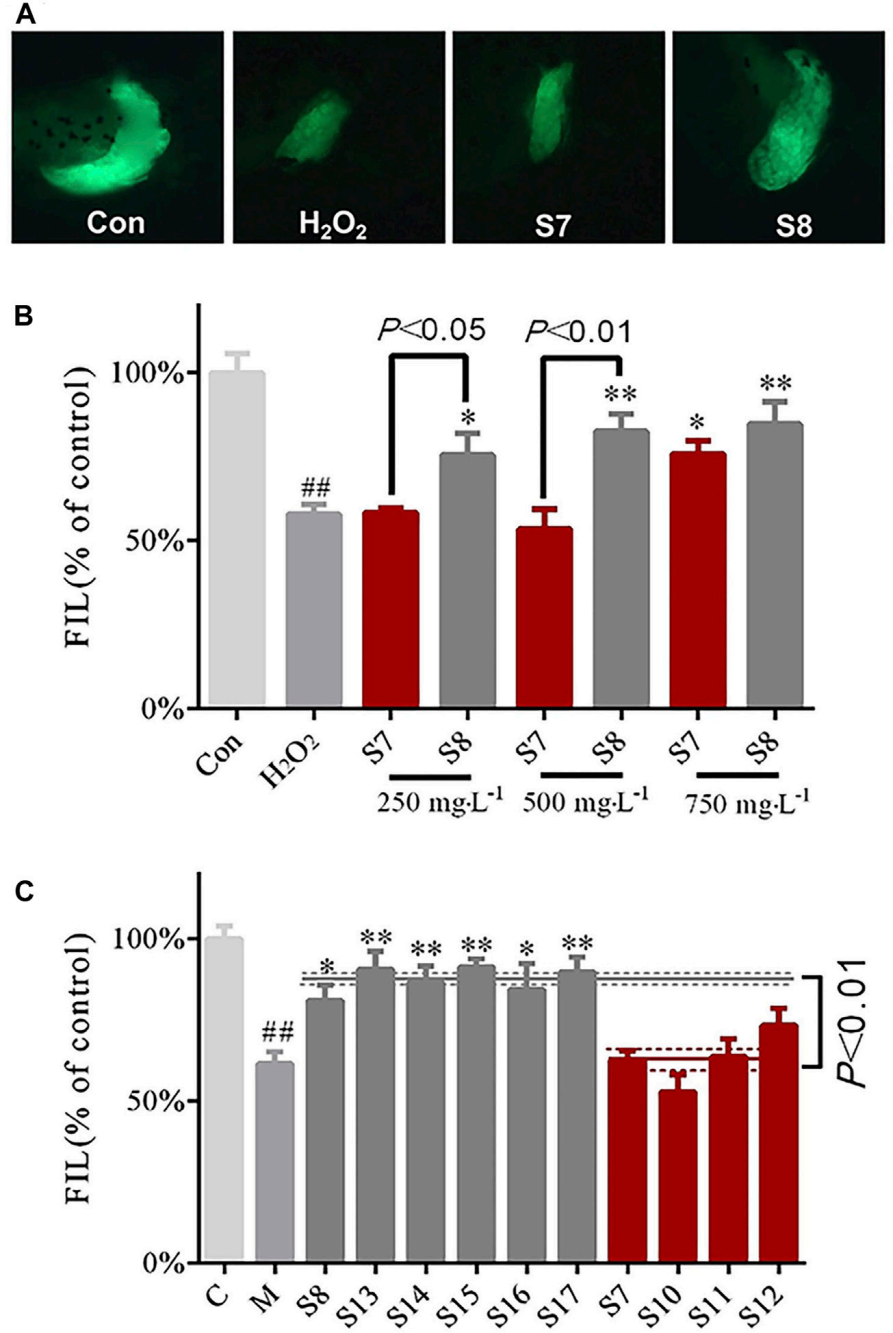
Comparison of the protective effects of GF (S7 and S8) collected from specific locations on liver injury **(A)** Phenotypic micrograph of the transgenic zebrafish with fluorescence label in the liver **(B)** Comparative analysis of protective effects of S7 and S8 with different doses on liver injury **(C)** Comparison of multiple batches of protective effects of GF from two areas, Fuding and Jiujiang, on the liver. *n* = 10.

**FIGURE 3:**
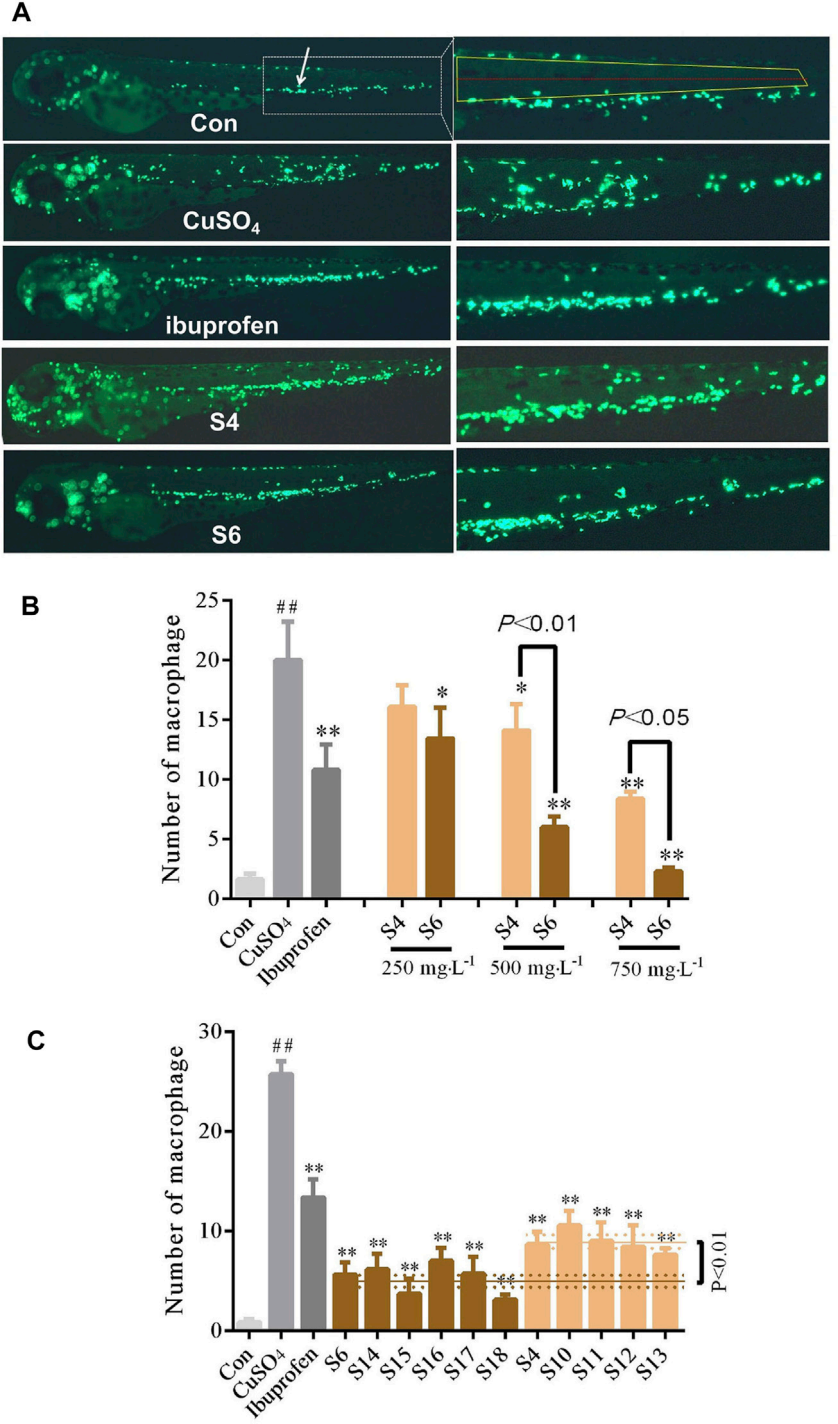
Comparison of the anti-inflammatory activities of GF (S4 and S6) collected from specific locations **(A)** Distribution phenotypic micrograph of neutrophils in zebrafish and enlarged drawing of neutrophils spreading to the lateral neuromast of the tail. at ×32 magnification **(B)** Comparative analysis of the effects of inflammation inhibition in zebrafish treated with S4 and S6 of different concentrations **(C)** Multiple batches’ comparison of anti-inflammatory effects of GF from two locations: Panan and Jichun. *n* = 10.

The protective effects of the GF samples S7 and S8 on liver injury were compared and verified at different concentrations (Figure 2B). The S4 and S6 GF samples were also evaluated using the zebrafish inflammatory model to identify the differences in their anti-inflammatory efficacies (Figures 3A,B).

The results showed that S7 GF differed statistically from the H_2_O_2_ model group at a concentration of 750 mgL^−1^; S8 GF differed statistically significantly from the H_2_O_2_ model group at concentrations of 250, 500 and 750 mgL^−1^. There were significant differences (*p* < 0.05) between S7 and S8 samples at concentrations of 250 and 500 mgL^−1^ (Figure 2B). When compared with the H_2_O_2_ model group, the six batches of GF from Fuding (S8, S13–S17) were statistically different, which indicates their protective efficacy against liver injury. The six batches of GF groups from Fuding and the four batches of GF groups from Jiujiang (S7, S10–S12) had significant differences (*p* < 0.01) (Figure 2C). These data further demonstrate that differences in the location of GF may be the main factor for the differences in the protective effects against liver injury.

The results demonstrated that S4 GF differed statistically significantly at concentrations of 500 and 750 mgL^−1^ from the CuSO_4_ model group; S6 GF was statistically significantly different at concentrations of 250, 500, and 750 mgL^−1^. There were statistical differences between S4 and S6 samples at concentrations of 500 and 750 mgL^−1^ (Figure 3B). When compared with the CuSO_4_ group, the five batches of GF from Panan (S4, S18-S21) and the six batches of GF from Jichun (S6, S22–S26) significantly inhibited the inflammation. There were statistically significant differences (*p* < 0.01) in samples obtained from these two locations (Figure 3C). This finding suggests that there are great variations in the biological activities of the GF samples sourced from different regions regardless of the time of collection.

### Discovery of the Bioactive Compounds in GF by Herbal Metabolomic Analysis

Ten batches of GF collected from Fuding and Jiujiang and 11 batches of GF collected from Panan and Jichun were analyzed using HPLC-Q-TOF/MS to obtain the original MS data. After being processed by MassHunter, the data were imported into R software to extract information on ion fragments. Finally, 1,056 component ion fragments were acquired from GF of Fuding and Jiujiang in the negative ion mode for further multivariate statistical analysis. In the same way, 926 component ion fragments were gained from GF of Panan and Jichun.

**FIGURE 4:**
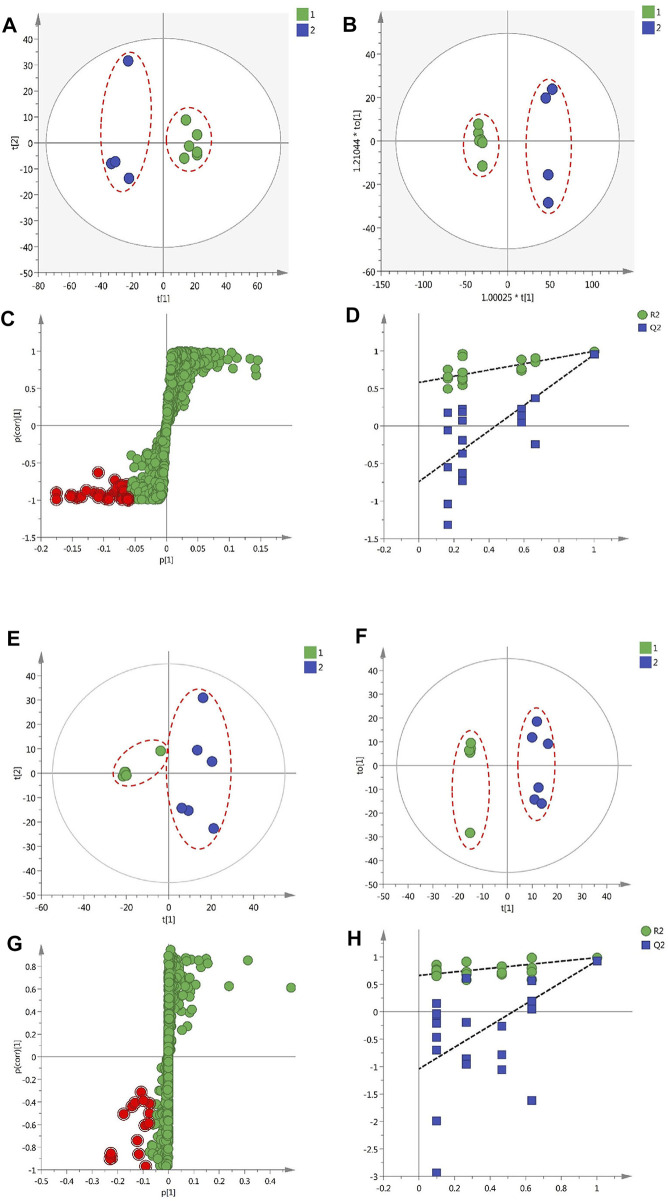
The results of multivariate statistical analysis of metabolomic data of protective effects of GF on liver injury and inflammation **(A)** A PCA scatter plot of 10 GF samples **(B)** The OPLA-DA scatter plot of the 10 samples **(C)** The S-plots for the OPLA-DA in the experiments of liver protection **(D)** PT test of the OPLA-DA model in experiments of liver protection **(E)** The PCA scatter plot of 11 GF samples **(F)** The OPLA-DA scatter plot of 11 samples **(G)** S-plots for the OPLA-DA in anti-inflammatory experiments **(H)** PT test of the OPLA-DA model in anti-inflammatory experiments.

In the PCA scatter plot of 10 GF samples from Fuding and Jiujiang in liver protection experiment (Figure 4A), the confidence interval was 95%, significance level was 0.05, and the first and second principal components were 53.1 and 15.3%, respectively. The results signified that GF in the two groups were clearly separated by the coordinate axis t [1]; PCA analysis was performed on the 926 common ion fragments obtained from the 11 GF batches from Panan and Jichun in the above-mentioned anti-inflammatory experiments. PCA plots (Figure 4E) were constructed directly using the first principal component (34.2%) and the second principal component 2 (23%), which showed that the GF samples from Panan and Jichun were clearly separated. The findings indicated that the chemical compositions of the two were significantly different.

Intra-group differences cannot be ignored and random errors cannot be eliminated by PCA, which is an unsupervized analysis method. The technique ignores the changing regulations and overall characteristics of the data, which is not conducive for the discovery of inter-group differences and metabolite differences (Hayashi et al., 2019). Therefore, this study used the supervised OPLS-DA model (Han et al., 2018) to further analyze the 1,056 and 926 common ion fragments obtained from 10 batches and 11 batches of GF, respectively. The OPLS-DA model can maximize the differences between the two groups of samples to determine the differences in the components between the two groups (F. Wang et al., 2019). From Figures 4B,F, it can be seen that the two groups of GF samples in the OPLS-DA model were clearly distinguished from each other, and the values of R_2_Y (cum) and Q_2_ (cum) for the models were 0.996 and 0.951 and 0.889 and 0.929, respectively. These results indicate that the OPLA-DA model has good applicability and predictability. The results of PT were calculated to be [R_2_= (0.0, 0.85), Q_2_ = (0.0, −0.47)] and [R_2_= (0.0, 0.98), Q_2_ = (0.0, −1.69)]. In addition, all the Q_2_ values on the left were less than the highest points on the right, and the intersection point of the blue regression line and ordinate of the Q_2_ value was less than zero (X. Zhang et al., 2019) (Figures 4D,H). This finding proves that the OPLS-DA model has good reliability. VIP (variables important in the projection) value >2, *p* < 0.05, and absolute value of the correlation coefficient |pcorr| > 0.58 were used to screen and determine the potentially important fragment ions. The results were represented using S-plots of the OPLA-DA model in the statistical analysis (Figures 4C,G). The red dots represent the filtered compounds. The protective effects of a total of 27 potentially important fragment ions against liver injury were acquired by filtering, and 21 fragment ions of potential anti-inflammatory activity components were identified (Table 2).

**TABLE 2:** Potential bioactive compounds of liver protection and anti-inflammatory activities of GF.

Bioactivity	Fragment ion	VIP	*p*	pcorr
Liver-protection	M775T741	5.56039	−0.4287	−0.89967
	M597T965	5.51121	−0.4282	−0.99089
	M1099T625	4.851	−0.37244	−0.95126
	M439T163	4.82011	−0.37208	−0.93084
	M595T625	4.54775	−0.35094	−0.97373
	M595T625	4.44954	−0.34427	−0.95797
	M663T625	4.27046	−0.331	−0.95091
	M551T1097	4.02831	−0.31189	−0.87264
	M695T1035	3.63873	−0.28251	−0.89842
	M723T387	3.4368	−0.26676	−0.96428
	M433T740	3.52129	−0.26558	−0.6328
	M776T741	3.36436	−0.25951	−0.90672
	M361T387	3.05187	−0.23623	−0.97732
	M598T965	2.91228	−0.22629	−0.98998
	M585T625	2.89894	−0.22512	−0.9482
	M612T625	2.70767	−0.20885	−0.90059
	M1089T1008	2.87941	−0.20018	−0.72838
	M447T739	2.50984	−0.19356	−0.83194
	M596T625	2.30536	−0.17825	−0.97174
	M809T1035	2.29357	−0.1739	−0.80255
	M475T387	2.22711	−0.17296	−0.98774
	M664T625	2.20186	−0.17058	−0.95159
	M552T1097	2.14931	−0.1663	−0.87771
	M550T625	2.1446	−0.16585	−0.95865
	M501T742	2.19594	−0.16549	−0.80683
	M696T1035	2.11204	−0.16389	−0.90292
	M1090T1008	2.14256	−0.15397	−0.78597
Anti-inflammatory	M391T391	2.07985	<0.01	−0.00652
	M725T1046	2.18122	<0.01	−0.01945
	M595T633	3.65777	<0.01	−0.02027
	M549T633	2.62687	<0.01	−0.03107
	M181T160	2.25526	<0.01	−0.09977
	M612T633	3.5166	<0.01	−0.09913
	M449T457	2.6484	<0.01	−0.41305
	M403T486	2.47847	<0.01	−0.49894
	M193T162	2.30153	<0.01	−0.58739
	M477T878	2.27194	<0.01	−0.96654
	M367T168	2.61018	<0.01	−0.60584
	M369T160	3.53082	<0.01	−0.38671
	M191T170	4.90403	<0.01	−0.3136
	M543T951	2.9708	<0.01	−0.8639
	M133T194	3.37134	<0.01	−0.73861
	M195T163	4.96994	<0.01	−0.40426
	M695T1037	5.08236	<0.01	−0.44032
	M449T486	5.73345	<0.01	−0.5096
	M353T677	5.7458	<0.01	−0.89675
	M609T950	5.93131	<0.01	−0.85521
	M209T164	5.82656	<0.01	−0.90237

### Results of GCA

GCA was used to analyze the spectral and effect relationships among the 27 fragment ions obtained from previous metabonomic analysis. The protective effects of GF sourced from the nine producing areas against liver injury and the 21 fragment ions and anti-inflammatory efficacies of GF were also examined. The gray relational degrees were calculated and sorted order respectively to verify the potential active ingredient (degree >0.7) (Table 3).

**TABLE 3:** Components of liver protection and anti-inflammatory effects of GF with gray relational degree>0.75 and component information.

Bioactivity	No.	Fragment ion	t_R_	m/z	Correlation	Compound identification	Chemical structure
Liver protection	1	M775T741	12.35	775.2638 [2M-H]^−^	0.79	Geniposide^a^	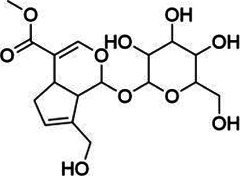
		M776T741	12.35	776.2676 [2M+1-H]^−^	0.8	Geniposide^a^
		M433T740	12.35	433.1341 [M + HCOO]^−^	0.73	Geniposide^a^
		M501T742	12.35	501.122 [M + CF_3_COO]^−^	0.74	Geniposide^a^	
	2	M663T625	10.41	663.1738 [M + CF_3_COO]^−^	0.75	Genipin-1-β-D-gentiobioside^a^	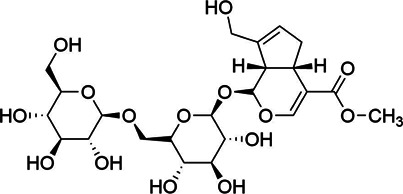
		M664T625	10.41	664.177 [M+1 + CF_3_COO]^−^	0.75	Genipin-1-β-D-gentiobioside^a^
		M595T625	10.41	595.1865 [M + HCOO]^−^	0.75	Genipin-1-β-D-gentiobioside^a^
		M596T625	10.41	596.1899 [M+1 + HCOO]^−^	0.74	Genipin-1-β-D-gentiobioside^a^
		M612T625	10.41	612.1769 [M + NO_3_]^−^	0.74	Genipin-1-β-D-gentiobioside^a^
		M549T625	10.41	549.1799 [M-H]^−^	0.73	Genipin-1-β-D-gentiobioside^a^
		M550T625	10.41	550.1832 [M+1-H]^−^	0.73	Genipin-1-β-D-gentiobioside^a^
		M585T625	10.41	585.1576 [M + Cl]^−^	0.72	Genipin-1-β-D-gentiobioside^a^	
	3	M1089T1008	16.8	1,089.361 [M + CF_3_COO]^−^	0.73	Crocin-1^a^	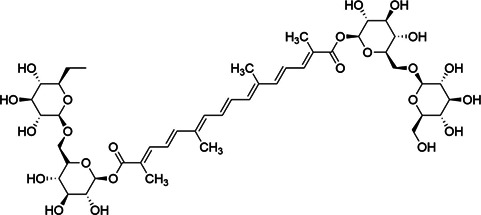
		M1090T1008	16.8	1,090.3650 [M+1 + CF_3_COO]^−^	0.73	Crocin-1^a^	
	4	M809T135	17.25	809.2104 [M + CF_3_COO]^−^	0.73	6″-O-*p*-coumaroyl-genipin-gentiobioside^b^	
		M695T135	17.25	695.2166 [M-H]^−^	0.71	6″-O-*p*-coumaroyl-genipin-gentiobioside^b^	
		M696T135	17.25	696.2211 [M+1-H]^−^	0.7	6″-O-*p*-coumaroyl-genipin-gentiobioside^b^	
	5	M361T387	6.45		0.76	Unkown	
Anti-inflammatory	6	M191T170	2.83	191.0559 [M-H]^−^	0.91	quinic acid^a^	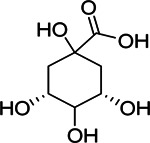
	7	M403T486	8.09	403.1217 [M-H]^−^	0.84	Gardenoside^a^	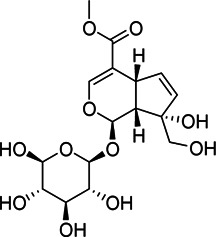
	8	M449T457	7.61	449.1290 [M-H]^−^	0.83	Feretoside^b^	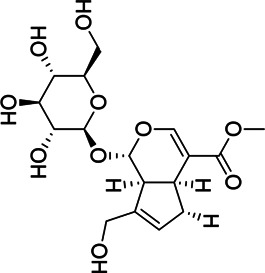
	9	M369T160	2.67	369.0675 [2M-H_2_O-H]^−^	0.92	D-glucuronic acid^a^	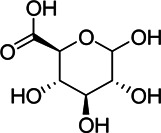
		M193T162	2.7	193.0355 [M-H]^−^	0.84	D-glucuronic acid^a^	
	10	M133T194	3.22	133.0143 [M-H]^−^	0.9	L-malic acid^a^	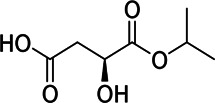
	11	M181T160	2.67	181.0718 [M-H]^−^	0.86	Mannitol^a^	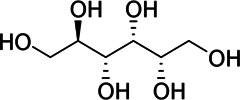
	12	M609T950	15.83	609.144 [M-H]^−^	0.88	Rutin^a^	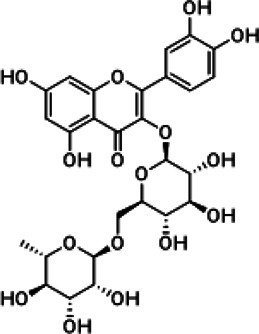
Anti-inflammatory	13	M209T164	2.73	209.0308 [M-H]^−^	0.8	D-glucaric acid^a^	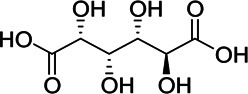
	14	M391T391	6.51	391.1237 [M-H]^−^	0.82	Shanzhiside^a^	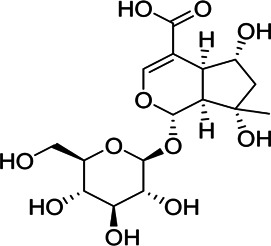
	15	M353T677	11.27	353.0867 [M-H]^−^	0.84	Chlorogenic acid^a^	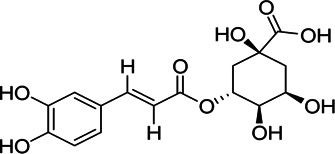
	2	M595T633	10.55	595.186 [M + HCOO]^−^	0.91	Genipin-1-β-D-gentiobioside^a^	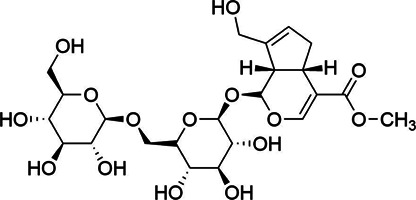
		M612T633	10.55	612.1763 [M + NO_3_]^−^	0.9	Genipin-1-β-D-gentiobioside^a^
		M549T633	10.55	549.1793 [M-H]^−^	0.89	Genipin-1-β-D-gentiobioside^a^	
	16	M543T951	15.84	543.2038 [M + CF_3_COO]^−^	0.88	10-O-acetylgeniposide^b^	
	17	M695T1037	17.28	695.2168 [M-H]^−^	0.88	6″-O-*p*-coumaroyl-genipin-gentiobioside^b^	
	18	M195T163	2.71	195.0511	0.86	Unkown	
	19	M367T168	2.8	367.0515	0.85	Unkown	

a refers to an identified component

b refers to a speculated component; Identification of active components according to MS data from reference (Y. Han et al., 2015; J. Zhou et al., 2020 and L.Wang et al., 2016).

### Identification of Active Components

**FIGURE 5:**
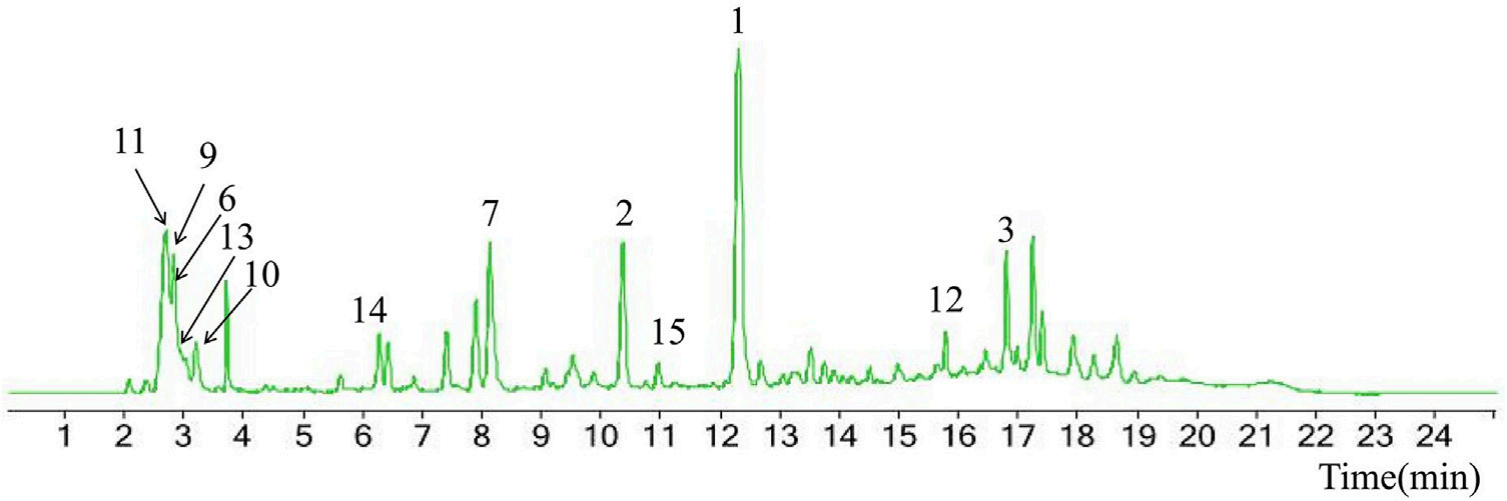
TICs of GF compounds at a negative ion mode. The total ion chrtograms (TICs) of GF extract, 1, 2, three on behalf of geniposide, genipin-1-β-D-gentiobioside, crocin-1, respectively; 6,7 and 9–15 represent quinic acid, gardenoside, d-glucuronic acid, l-malic acid, mannitol, rutin, d-glucaric acid, shanzhiside, chlorogenic acid, and genipin-1-β-D-gentioside, respectively.

Chemical information related to the GF components is provided in Table 3. The mass numbers of four components related to liver protection and 15 components related to anti-inflammatory effect were analyzed qualitatively. After quality measured by MS compared with the MS database of GF chemical composition, possible compounds were identified. Based on the retention time and m/z data from the total ion chromatograms (TICs) of GF compounds, geniposide (1), genipin-1-β-D-gentiobioside (2), and crocin-1 3) were identified as the pharmacodynamic compounds offering liver protection. In addition, component 4 was presumed to be 6″-O-*p*-coumaroyl-genipin-gentiobioside, and component 5 was not recognized (Figure 5A); The anti-inflammatory components were finally identified as quinic acid (6), gardenoside (7), d-glucuronic acid (9), l-malic acid (10), mannitol (11), Rutin (12), d-glucaric acid (13), shanzhiside (14), chlorogenic acid (15), and genipin-1-β-D-gentioside (2); Furthermore, due to the large error of component 3, it could only be inferred as feretoside. Components 16 and 17 were presumed to be 10-O-acetylgeniposide and 6″-O-*p*-coumaroyl-genipin-gentiobioside, and components 18 and 19 were not confirmed (Figure 5B).

### Confirmation of the Identified Bioactive Compounds

**FIGURE 6:**
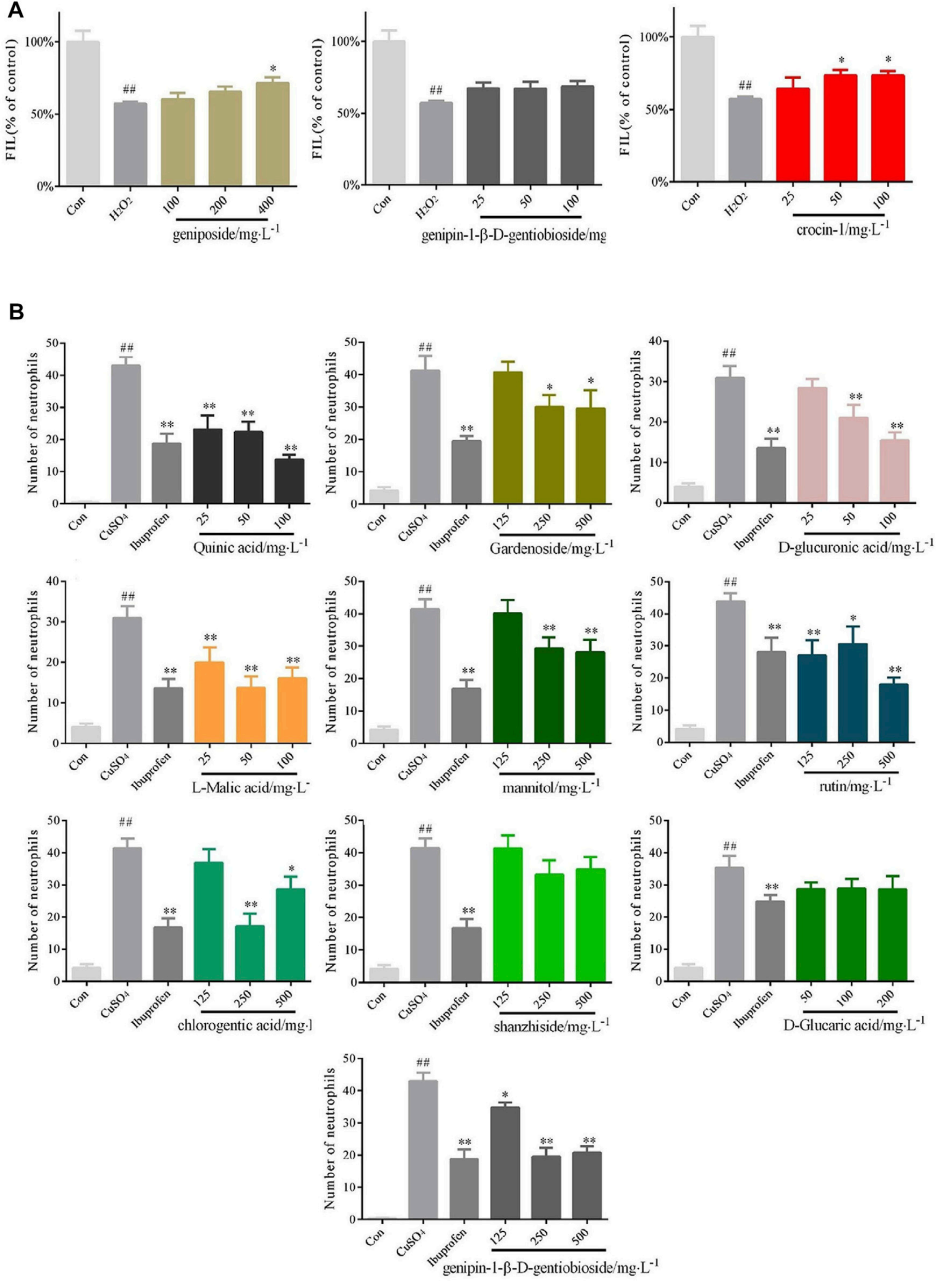
Confirmation of the protective effects of the identified bioactive compounds of GF on liver injury and inflammation **(A)** Quantified statistical results of protective effects of geniposide, genipin-1- β-D-gentiobioside, and crocin-1 with different doses on liver injury. n = 10 **(B)** Quantified statistical results of anti-inflammatory effects of quinic acid, gardenoside, d-glucuronic acid, l-malic acid, mannitol, rutin, chlorogenic acid, shanzhiside, d-glucaric acid, and genipin-1-β-D-gentioside with different doses. *n* = 10.

The zebrafish model of liver injury was applied to verify whether the above three identified components (geniposide, genipin-1-β-D-gentiobioside and crocin-1) can be used as chemical markers for the protective effects of GF on the liver. The results suggested that the group administrated with genipin-1-β-D-gentioside did not statistically differ from the H_2_O_2_ model group; hence, the compound did not exhibit a protective effect. The groups administrated with geniposide at concentrations of 400 mgL^−1^ and crocin-1 at concentrations of 50 and 100 mgL^−1^ were statistically different from the H_2_O_2_ model group, which proved that the two components offered liver protection (Figure 6A). The zebrafish model of inflammation was applied to verify whether the 10 identified chemical components can be used as anti-inflammatory markers. The results showed that when compared with the model group, shanzhiside and d-glucaric acid did not inhibit inflammation. Thus, the two components cannot be used as anti-inflammatory markers. On the other hand, quinic acid and l-malic acid both differed statistically significantly at concentrations of 25, 50, and 100 mgL^−1^ from the CuSO_4_ model group; Rutin was statistically significantly different at concentrations of 125, 250, and 500 mgL^−1^; There were significant differences (*p* < 0.01) between d-glucuronic acid and the CuSO_4_ model group at concentrations of 50 and 100 mgL^−1^; Gardenoside, mannitol, chlorogenic acid and genipin-1-β-D-gentiobioside at concentrations of 250 and 500 mgL^−1^ inhibited inflammation to varying degrees (Figure 6B).

## Discussion

According to ancient Chinese medical book’s record, the traditional efficacy of GF can be classified into two main categories: one is clearing *Heat* and the other is purging *Fire*. According to modern pharmacology, the effect of “purging *Fire*” is closely related to liver and gallbladder systems. At present, GF is mainly used to treat two types of diseases: 1. dampness–heat jaundice (J. Y. Li et al., 2017) such as epidemic or iteric hepatitis, jaundice, fever, and short red urine caused by stagnation of hepatochlic hygropyrexia; 2. eye redness, swelling, and pain (Chen et al., 2020), caused by hepatobiliary fire attack and other eye discomforts. These two kinds of diseases are related to liver injury. Modern research has confirmed that decoction and alcohol extract of GF have choleretic effect, promote bile secretion, and reduce the bilirubin in the blood to promote its rapid excretion (Tian et al., 2017). Seo (Seo et al., 2017) found that genipin, the main component of GF, ameliorates galactosamine/lipopolysaccharide (LPS)-induced hepatocellular damage by suppressing the signal transduction of necroptosis-mediated inflammasome to exert hepatoprotective effects. Therefore, we considered that the protective effect of GF against liver injury in the zebrafish model can be applied as one of the evaluation indexes of the efficacy of GF. On the other hand, the effectof “clearing *Heat*” of GF is closely related to its anti-inflammatory properties (Q. Wang et al., 2018). GF is mainly used to treat inflammation such as heat toxin sores and eye redness, swelling, and pain. Besides GF suppresses *Streptococcus hemolyticus*, dermatophytes, and viral replication (Kim et al., 2020). Inflammatory response caused by bacterial and fungal infections is the chief manifestation of infectious diseases. By exploring the molecular mechanism of geniposide in the mouse macrophages inflammation model caused by LPS, Shi et al. (Q. Shi et al., 2014) determined that geniposide could inhibit the release of inflammatory factors such as tumor necrosis factor-alpha (TNF-α) and interleukin-6 (IL-6). Besides, geniposide can alter the level of mRNA and the key enzymes cyclooxygenase-2 and inducible isoform of nitric oxide synthase in the inflammatory response. Therefore, it was believed that clearing heat and detoxifying, the main clinical effects of GF, had a relatively close relationship with anti-inflammatory properties. Hence, the anti-inflammatory model of zebrafish was selected to characterize the heat-clearing effect of GF.

The genome of zebrafish has been proved a high homology with that of humans (MacRae and Peterson, 2015). In this work, the zebrafish model of liver injury induced H_2_O_2_ was applied to evaluate the protective effects of GF on the liver. As a strong oxidant, H_2_O_2_ can cause apoptosis outside the cell and promote the production of hydroxyl free radicals, thereby leading to cell death induced by the lipid peroxidation of the cell membrane (B. Huang et al., 2018). In this study, zebrafish was treated with 25 μL L^−1^ hydrogen peroxide for 2 days. There was a significant difference between the H_2_O_2_ group and the control group, which proved that the liver injury model of zebrafish was successfully established and could be used for the discovery of hepatoprotective pharmacodynamic markers of GF. The defense system of zebrafish is highly conservative and has a complete response system similar to that of humans, including the migration and phagocytosis of the inflammatory cells (Robertson et al., 2016). Inflammation caused by CuSO_4_ in zebrafish belongs to the category of drug-induced reaction among the various inflammatory factors. Zhang et al. (Y. Zhang et al., 2020). utilized the inflammation model of zebrafish induced by CuSO_4_ to study the anti-inflammatory mechanism of isoniazid. Transgenic zebrafish with fluorescence-labeled neutrophils used in this study demonstrated the phenotypic process of inflammation. Several neutrophils spread close to the lateral neuromast in the zebrafish after treatment with CuSO_4_. After zebrafish larvae was given 4 mg L^−1^ copper sulfate for 1 h, there was a significant difference between the CuSO_4_ group and the control group, which proved that the zebrafish inflammation model was successfully established and could be used for the discovery of anti-inflammatory pharmacodynamic markers of GF.

In this work, representative samples of GF from different parts of China were collected to study the relationship between the composition of GF and its bioactivity. We conceived and established a new strategy of “omics discrimination-grey correlation-biological verification”. In this strategy, herbal metabolomics was used for high-resolution omics detection and analysis. The chemometrics method of gray correlation was employed to establish the correlation between the components and the activity. The zebrafish model was utilized to evaluate the activity of batches of TCM samples and verify the activity of the monomer components. The difficulty of bioactivity association of trace components of TCM was solved by this strategy. When compared with the existing techniques, the speed of research was greatly accelerated and the steps were simplified, which had obvious advantages.

The conventional view is that only the components which can enter the bloodstream is likely true pharmacodynamic substances of TCM. So whether with the potential of entering the bloodstream can be considered as an index for judging them as Q-Marker. Present studies have shown that at least 35 extracted components were detected in the bloodstream when GF was administered to animals (X. Zhang et al., 2018). Among the 10 components (geniposide, genipin-1-β-D-gentiobioside, crocin-1, quinic acid, gardenoside, d-glucuronic acid, l-malic acid, mannitol, rutin, and chlorogenic acid) examined for protective effects against liver injury and inflammation in this zebrafish experiment. Among them, geniposide, genipin-1-β-D-gentiobioside, crocin-1, gardenoside, rutin, and chlorogenic acid were found to significantly migrate to the blood (R. Zhang et al., 2020).

In this research, geniposide and crocin-1 were discovered as hepatoprotective pharmacodynamic markers of GF. On the other hand, quinic acid, gardenoside, d-glucuronic acid, l-malic acid, mannitol, rutin, chlorogenic acid, shanzhiside, d-glucaric acid, and genipin-1-β-D-gentioside were also confirmed as anti-inflammatory pharmacodynamic markers of GF. Some studies have reported that geniposide has significant hepatoprotective effects and gardenoside has anti-inflammatory effects. Zhang et al. (T. Zhang et al., 2016). found that geniposide can alter the abnormal liver metabolism caused by ethanol exposure, thereby alleviating the disturbance of amino acid metabolism and oxidative stress. Wang et al. (J. Wang et al., 2016). stated that geniposide can protect mice from liver injury caused by tripterygium glycosides as it alleviates oxidative stress and inflammation and promotes tissue repair and regeneration. Liang et al. (Liang et al., 2015) indirectly clarified that gardenoside may inhibit the production of inflammatory cytokines TNF-α, IL-1β, and IL-6 while exploring the effects of gardenoside on the steatosis of HepG2 cells induced by free fatty acids. Interestingly, we found that genipin-1-β-D-gentiobioside has a strong anti-inflammatory effect, which has not been previously reported in the literature. This effect warrants in-depth study to gather more evidence for the heat-reducing effect of GF. The iridoid of GF derived from Gardenia Ellis belonging to the Rubiaceae family is considered to be a representative component and a crucial chemical marker. Gardenoside, geniposide and genipin-1-β-D-gentioside are the main components of iridoids. Among them geniposide is the highest content. Quinic acid, d-glucuronic acid, l-malic acid, mannitol and chlorogenic acid belong to organic esters of GF, among which chlorogenic acid is its representative component. Rutin is the only flavonoid with anti-inflammatory activity in the results of this study (W. Li et al., 2021; Xiao et al., 2017). Therefore, based on the factors such as pharmacodynamic correlation, herbal specificity, and measurability, we believe that geniposide, genipin-1-β-D-gentiobioside, and gardenoside have good representativeness and can reflect the intrinsic quality of GF. Hence, they were identified to be the quality markers of GF.

## Conclusion

In summary, we have devised a new strategy to study the Q-Markers of TCM with hepatoprotective and anti-inflammatory effects. In this research, through the effective integration of herbal metabolomics, zebrafish models, chemometrics, and other technologies, we have quickly identified “markers” that can reflect the intrinsic quality of TCM. We have also conducted research on the key pharmacodynamic components of GF and have proposed a combination of Q-Markers of GF, which provides the scientific basis for quality evaluation, resource utilization, and standard establishment of GF.

## Data Availability

The raw data supporting the conclusion of this article will be made available by the authors, without undue reservation, to any qualified researcher.
