# Erratum: An Integrated Strategy for Rapid Discovery and Identification of Quality Markers in Gardenia Fructus Using an Omics Discrimination-Grey Correlation-Biological Verification Method

**DOI:** 10.3389/fphar.2021.742055

**Published:** 2021-07-27

**Authors:** Frontiers Production Office

**Affiliations:** Frontiers Production Office, Frontiers Media SA, Lausanne, Switzerland

**Keywords:** gardenia fructus, zebrafish, quality markers, herbal metabolomics, gray correlation analysis, anti-inflammatory, liver protection

Due to a production error, there was an error in affiliation 1. Instead of “Shandong Medical University and Shandong Academy of Medical Science”, it should be “Shandong First Medical University and Shandong Academy of Medical Sciences”.

The publisher apologizes for this mistake. The original version of this article has been updated.

